# Exploring the Synergy between Cellobiose Dehydrogenase from *Phanerochaete chrysosporium* and Cellulase from *Trichoderma reesei*

**DOI:** 10.3389/fmicb.2016.00620

**Published:** 2016-04-29

**Authors:** Min Wang, Xuefeng Lu

**Affiliations:** ^1^Key Laboratory of Biofuels, Qingdao Institute of Bioenergy and Bioprocess Technology, Chinese Academy of SciencesQingdao, China; ^2^Shandong Provincial Key Laboratory of Energy Genetics, Qingdao Institute of Bioenergy and Bioprocess Technology, Chinese Academy of SciencesQingdao, China; ^3^Shandong Provincial Key Laboratory of Synthetic Biology, Qingdao Institute of Bioenergy and Bioprocess Technology, Chinese Academy of SciencesQingdao, China

**Keywords:** *Tritroderma reesei*, cellulase, cellobiose inhibition, cellobiose dehydrogenase, synergy

## Abstract

Recent demands for the production of lignocellulose biofuels boosted research on cellulase. Hydrolysis efficiency and production cost of cellulase are two bottlenecks in “biomass to biofuels” process. The *Trichoderma* cellulase mixture is one of the most commonly used enzymes for cellulosic hydrolysis. During hydrolytic process cellobiose accumulation causes feedback inhibition against most cellobiohydrolases and endoglucanases. In this study, we demonstrated the synergism effects between cellobiose dehydrogenase (CDH) and cellulase both *in vitro* and *in vivo*. The CDH from *Phanerochaete chrysosporium* was heterologously expressed in *Pichia pastoris*. Supplementation of the purified CDH in *Trichoderma* cellulase increased the cellulase activities. Especially β-glucosidase activity was increased by 30–100% varying at different time points. On the other hand, the *cdh* gene was heterologously expressed in *Trichoderma reesei* to explore the synergism between CDH and cellulases *in vivo*. The analyses of gene expression and enzymatic profiles of filter paper activity, carboxymethylcellulase (CMCase) and β-glucosidase show the increased cellulase activity and the enhanced cellulase production in the *cdh*-expressing strains. The results elucidate a possible mechanism for diminishing the cellobiose inhibition of cellulase by CDH. These findings provide a novel perspective to make more economic enzyme cocktails for commercial application or explore alternative strategies for generating cellulase-producing strains with higher efficiency.

## Introduction

The filamentous fungus *Trichoderma reesei* produces a set of enzymes that cooperate synergistically in the degradation of cellulose. They are nowadays a paradigm for commercial scale production of cellulases and employed for the saccharification of cellulose biomass to simple sugars for biofuel production. The widely accepted mechanism for enzymatic cellulose hydrolysis involves synergistic actions by endoglucanase (EGs) (EC 3.2.1.4), exoglucanase (cellobiohydrolases, CBHs, in the case of *T. reesei*) (EC 3.2.1.91; EC 3.2.1.176), and β-glucosidases (BGLs) (EC 3.2.1.21) (Zhang and Lynd, 2004). Endoglucanases randomly hydrolyze accessible intramolecular β-1,4-glucosidic bonds of cellulose chains to produce new chain ends; exoglucanases processively cleave cellulose chains at the ends to release soluble cellobiose or glucose; and β-glucosidases hydrolyze cellobiose to glucose. These three hydrolysis processes occur simultaneously.

The mechanism of induction and repression on cellulase stimulated by hydrolysates formed from cellulose hydrolysis has been explored in depth (Schmoll and Kubicek, 2003). In most cases, the production and/or activity of cellulase components are repressed or inhibited by the hydrolysis products (Holtzapple et al., 1990; Ju and Afolabi, 1999). It is well established that cellobiose with high concentration inhibits the activity of cellulase (George et al., 2001). It is a stronger inhibitor than glucose (Holtzapple et al., 1990). Accumulation of cellobiose causes feedback inhibition against most cellobiohydrolases and endoglucanases, and thus it becomes important to supply enough β-glucosidases to hydrolyze cellobiose to glucose in order to eliminate such inhibition.

In *T. reesei* cellulase system, the two most abundant proteins are cellobiohydrolases CBH I and CBH II, which are known to account for 70 to 80% of the total cellulases (Nummi et al., 1983). The other abundant component is endoglucanase EG II (CEL5A) with up to 15%. β-glucosidases is relatively scarce in *T. reesei*. A concept of designing a highly efficient cellulase mixture for hydrolysis of cellulose has been reported (Gusakov et al., 2007), and inspired many efforts to improve cellulase performance by making the cocktails with supplementary β-glucosidases and/or other components (Knauf and Moniruzzaman, 2004). β-glucosidases from *Aspergillus niger* has been purified and manufactured as the commercial Novozyme 188 (Novozymes), which is one of the most commonly used enzyme for promotion of a synergistic effect with *Trichoderma* cellulases. The cocktail formulation supplemented with this BGL can efficiently produce fermentable glucose and the hydrolytic efficiency was increased by enhancing the β-glucosidase activity in the enzyme cocktail (Ma et al., 2011; Dashtban and Qin, 2012; Nakazawa et al., 2012). The synergism between *A. niger* BGL and *T. reesei* cellulase has triggered interest in screening and discovery other promise enzymes to create more effective and balanced commercial cellulase mixtures (Park et al., 2012).

Cellobiose dehydrogenase (CDH, EC 1.1.99.18) is an extracellular enzyme produced by various wood-degrading fungi (Henriksson et al., 2000). It oxidizes the reducing end of cellobiose and cellooligosaccharides to their corresponding 1, 5-lactones, which are subsequently hydrolyzed to the carboxylic acids. Early in 1978, Ayers suggested that CDH (named as cellobiose oxidase, CBO back then) from *Sporotrichum pulverulentum* (*Phanerochaete chrysosporium*) might be involved in cellulose biodegradation (Ayers et al., 1978). Bao and Renganathan (1992) reported that CBO which was purified from extracellular medium of *Phanerochaete chrysosporium* could enhance crystalline cellulose degradation by *T. viride* and *T. reesei* cellulases, but the mechanism remained unclear. Igarashi demonstrated the synergistic interaction between CDH and CBH I from *P. chrysosporium* (Igarashi et al., 1998). Decomposition of bacterial cellulose microcrystalline by CBH I was enhanced by the combined addition of CDH/ ferricyanide. The reason was clarified that CDH could relieve the competitive inhibition of cellobiose to CBH I by its oxidation to cellobionolactone. The synergism between CDH and CBH I observed in *P. chrysosporium* inspired our interests in application of this synergism in *T. reesei* cellulase system.

The synergism of CDH from another wood-rotting basidiomycete, *Pycnoporus cinnabarinus*, with *T. reesei* industrial enzyme cocktail in the saccharification process on wheat straw has been shown by Bey et al. (2011) who found that CDH increased the saccharification process due to gluconic acid production. In this work, we studied the synergism between CDH and CBHs, EGs, and β-glucosidases both *in vitro* and *in vivo*. The CDH from *Phanerochaete chrysosporium* was used to further diminish cellobiose inhibition to cellulases. Supplementation of the purified CDH in *Trichoderma* cellulase increased the cellulase activity, especially β-glucosidase activity *in vitro*. Moreover, the *cdh* gene was heterologously expressed in *T. reesei* QM9414 under the control of *cbh2* promoter and produced a recombinant version of cellulase mixture. The cellulase production and the hydrolysis efficiency of *cdh*-expressing *Trichoderma* transformants were significantly increased.

## Materials and Methods

### Construction of *cdh* Expression Vector in *P. pastoris*

The *cdh* gene (GenBank Accession U50409) from *Phanerochaete chrysosporium* was synthesized in Sangon Biotech (Shanghai, China) and cloned into pUC57, yielding the plasmid pc-cdh.

The native signal peptide and stop codon of the *cdh* gene were removed by PCR with the forward primer mcdh-Ep1 (*Eco*R I) and reverse primer mcdh-Xbp2H (*Xba* I) (Supporting Information Table S1). The plasmid pc-cdh was used as the template. The PCR products were digested with *Eco*R I and *Xba* I and inserted into pPICZαA at *Eco*R I and *Xba* I sites, yielding the plasmid pWM79.

### Transformation and Isolation of *P. pastoris* GS115 Transformants

The linearized pWM79 was transformed to *P. pastoris* GS115 by electroporation according to the manufacturer of BTX ECM630 electroporation system (NatureGene Corp.). The vector pPICZαA without insert was used as a control. The transformants were first screened on YPDS plates with different concentrations of zeocin (200–1000 μg/mL), and were further screened in minimal dextrose (MD) plates and methanol (MM) plates according to the *Pichia* Expression Kit manual (Invitrogen).

Small-scale expression in 20 mL BMMY medium and following SDS-PAGE and activity assay were used to confirm the recombinant *Pichia* clones expressing the correct CDH protein. Zeocin-resistant transformants were inoculated in 10 mL of BMGY medium (in 50 mL Erlenmeyer flask) at 28°C in a rotary shaker (200 rpm) for 12–16 h to an OD600 of 2, and the expression was induced by transferring cells into 20 mL of BMMY growing for a further 96 h. The medium was sampled and supplemented with 2% (v/v) methanol every 12 h. SDS-PAGE of culture supernatants at different time points was used to identify the optimal induction time and to determine which transformant had the best secretion yield. Scale-up expression was performed in 1 liter Erlenmeyer flask on a rotary shaker (250 rpm) at 28°C for 96 h in 200 mL medium.

### Purification and Characterization of the Recombinant CDH

After induction for 96 h, the medium was separated from the cells by centrifugation and proceed to purification. The culture supernatant was first concentrated using Amicon Ultra-15 (MWCO 30 kD, Millipore). The concentrated supernatant was dialyzed against Binding buffer and loaded on a His∙Bind Resin, Ni-charged column (Novagen). The purification was according to the His∙Bind^®^ Kits manual. The His-tagged CDH was eluted with Washing Buffer and dialyzed against PBS buffer. The protein was concentrated with Amicon Ultra-15. The concentration of the purified CDH was measured by the Bradford protein assay kit (Beyotime) with bovine serum albumin (BSA) as standard. The deglycosylation of the CDH protein was performed by Endo Hf (NEB).

Cellobiose dehydrogenase activity was determined by monitoring the reduction of 100 μM cytochrome *c* in 50 mM acetate buffer, pH 4.5 containing 1 mM cellobiose. The decrease in absorption at 550 nm was monitored at 25°C for 2 min. The *K*_M_ for cellobiose was determined at 25°C in 50 mM acetate buffer, pH 4.5 using cytochrome *c* as electron acceptor. The concentration of cellobiose ranged from 0.004 to 10 mM. Graphpad prism 5 was used for the non-linear regression calculation and kinetic parameter determination.

### Supplementation of the Purified CDH to Cellulase *in vitro*

QM9414 were pre-cultured in 250 mL Erlenmeyer flasks on a rotary shaker (200 rpm) at 30°C for 24 h in 50 mL Mandels-Andreotti (MA) medium supplemented with 1% (w/v) D-glucose for growth and 0.45% (w/v) lactose for initial inducer of cellulase production. Pre-grown mycelia were resuspended in 50 mL fermentation medium and incubated in 250 mL Erlenmeyer flasks on a rotary shaker (200 rpm) at 30°C for 48, 72, and 96 h. Fermentation medium was composed of MA medium, 1% (w/v) D-glucose and 2.6% (w/v) lactose (Eventhough glucose is reported to be a repressor of cellulase activities, from optimization experiments, its addition in the medium was contributed to the mycelia growth. The level of residual glucose in the medium reduced to 0.1% after 24 h). Culture supernatants were removed and collected by filtration. The purified CDH was added to the culture supernatants before cellulase activity analysis and the mixture was incubated at room temperature for 5 min to disperse uniformly. For comparison of the synergism effects between CDH and cellulase with the synergism effects between β-glucosidase and cellulase, the commercial glucosidase from *Aspergillus niger* (obtained from Sigma–Aldrich) (Ag-Bgl) and a recombinant thermostable β-glucosidase from *Thermatoga maritima* (Tm-BglA) (Song et al., 2011) were used to test the synergism effects between β-glucosidases and *Trichoderma* cellulase.

### Construction of Pcbh2-cdh Expression Cassette

The plasmid pSMZ1 bearing the promoter and terminator of the *cbh2* gene, was generously provided by Professor Christian P. Kubicek from Institute of Chemical Engineering, Vienna University of Technology (Zeilinger et al., 1998). The pSMZ1 was digested with *Not* I, then blunted and digested again with *Xba* I to get a blunt/*Xba* I vector backbone. The plasmid Pc-cdh containing the *cdh* gene, was digested with *Bam*H I, then blunted and digested again with *Xba* I. The resultant blunt/*Xba* I *cdh* fragment was ligated into blunt/*Xba* I pSMZ1 to yield plasmid pWM78. The Pcbh2-cdh expression cassette, a 4-kbp *EcoR* I/*Hind* III fragment from pWM78, was used to transform *T. reesei* QM9414. Transformation was carried out as described by Gruber et al. (1990) using co-transformation of pSMZ1. Transformants were selected with Hygromycin B resistance and the integration of the *cdh* gene into the genome was confirmed by genomic PCR. In order to avoid the homologous recombination in *cbh2* site, the genome PCR for the *cbh2* gene was used to confirm that the *cdh* expression cassette and Hygromycin B select cassette were not homologously recombined in the genome. The purified transformants were further selected on cellulose Cong Red Medium.

The screened cellulase producers were inoculated in liquid medium. A total of 10^8^ conidia per liter were used as inoculum. Mycelia were pre-cultured in 250 mL Erlenmeyer flasks on a rotary shaker (200 rpm) at 30°C for 24 h in 50 mL MA medium supplemented with 1% (w/v) D-glucose for growth and 0.45% (w/v) lactose for initial inducer of cellulase production. Pre-grown mycelia were resuspended in 50 mL fermentation medium and incubated in 250 mL Erlenmeyer flasks on a rotary shaker (200 rpm) at 30°C for 48, 72, and 96 h. Fermentation medium was composed of MA medium, 1% (w/v) D-glucose and 2.6% (w/v) lactose. For the experiments using cellulose as carbon source, pre-grown mycelia were resuspended in MA medium supplemented with 1% (w/v) microcrystalline cellulose as sole carbon source. The experiments were conducted in triplicate for each sample. After induction, mycelia were collected by filtration, washed, frozen in liquid nitrogen, stored at -80°C, and used for total RNA extraction and quantitative real-time PCR analysis. Culture supernatants were used for protein content detection and cellulase activities assays.

### RNA Extraction and Transcript Analysis by Quantitative Real-time PCR (qRT-PCR)

Total RNA was extracted using TRIZOL reagent (Invitrogen, USA) according to the manufacturer’s instructions. RNA was quantified by NanoVue (GE Healthcare), and the integrity was checked by gel electrophoresis in 1% agarose. Total RNA (1 μg) from each pooled sample was first digested with DNase I (TaKaRa) to remove genomic DNA. Synthesis of cDNA from total RNA was carried out using a cDNA Synthesis kit (TaKaRa) according to the manufacturer’s instructions. First-strand cDNA was synthesized from the same amount of total RNA (1 μg). Synthesized cDNA was diluted 1:20 and used as a template for quantitative real-time PCR. Reactions were performed in the LightCycler^®^ 480 System (Roche) using FastStart Universal SYBR Green Master ROX (Roche) for detection according to the manufacturer’s instructions. Actin transcript was used as an internal reference to normalize the amount of total RNA present in each reaction (Verbeke et al., 2009). The fluorescence threshold value was calculated using LightCycler^®^ 480 system software. The specificity of the PCR amplifications was documented by melting curve analysis. The expression levels of genes were calculated from the threshold cycle according to the 2 ^-ΔΔCT^ method (Livak and Schmittgen, 2001) relative to transcription levels of QM9414 (Liu et al., 2016). All assays were performed in triplicate with water as a negative control instead of cDNA. Gene-specific primers (Supporting Information Table S1) used in qRT-PCR were designed to amplify 150–200 bp of the internal coding region of each gene. Plasmid cDNA standards are also listed in Supplementary Table S1.

### Total Protein Concentration and Biomass Determination

Total protein concentrations in the culture supernatants were measured by the Bradford protein assay kit (Beyotime) with BSA as standard. For fermentation culture, biomass concentrations were determined by gravimetric analysis. In each time point, mycelia of 20 mL culture were collected by filtration. The mycelia were washed with ddH_2_O to get rid of salts and filtrated again. The pellets were dried overnight in an oven (70°C) to constant weight. For cellulose culture, biomass concentrations were indirectly measured by the amount of intracellular protein quantified by the Bradford protein assay kit (Beyotime) with BSA as standard. The results are means of three independent experiments cultivations.

### Cellulase Activity Assay

All samples were analyzed in triplicate and mean values were calculated. Overall cellulase activity of the samples was measured as Filter Paper activities (FPA) using the IUPAC-recommended procedure (Ghose, 1987). Endoglucanase activity was assayed as CMCase activity with carboxymethylcellulase (CMC) as substrate in 50 mM acetate buffer (pH 4.8) for 15 min at 50°C. For both activities, sugar release was assayed via the dinitrosalicylic acid (DNS) method using glucose as the standard. β-glucosidase activity was determined using 4-nitrophenyl-β-D-glucopyranoside with paranitrophenol as the standard (Berghem and Pettersson, 1974).

### Statistical Analysis

The statistical significance analyses of all tests were performed with one-way ANOVA followed by the Bonferroni test available in the GraphPad Prism 5.

## Results

### Heterologous Expression and Enzymatic Characterization of CDH

Here the *cdh* gene from *P. chrysosporium* was chosen as its previous profound investigation. The cDNA encoding CDH from *P. chrysosporium* has already been cloned by Raices and expressed in *P. pastoris* KM71 by Yoshida et al. (2001) with the expression vector pPIC9K. In this work, the *cdh* gene was heterologously expressed in *P. pastoris* GS115 with the expression vector pPICZαA. The *cdh* gene was synthesized and cloned into pUC57, yielding the plasmid pc-cdh.

**FIGURE 1:**
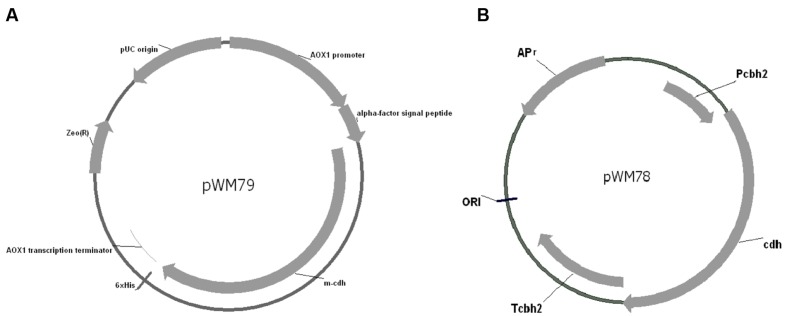
**Map of pWM79 and pWM78. (A)** Map of the plasmid pWM79 that constructed for transformation of *Pichia pastoris* GS115. **(B)** Map of the plasmid pWM78 that constructed for transformation of *Trichoderma reesei* QM9414.

The TMpred program predicted a membrane-spanning region in the CDH protein amino acid sequence. SignalP 4.1 program (Petersen et al., 2011) prediction reveals that the location of signal peptide cleavage site is in the 18th amino acid. The expression vector pPICZαA (Invitrogen), containing the α-factor secretion signal, allows high-level expression of the target gene in *Pichia*. In order to construct the *cdh* gene in pPICZαA in frame with the α-factor secretion signal and the C-terminal 6 × His tag, the native signal peptide and the stop codon of the *cdh* gene were removed by PCR. The digested PCR products were inserted into pPICZαA at the corresponding sites, yielding the plasmid pWM79 (**Figure 1A**).

The recombinant gene was then introduced into the *Pichia* genome under the control of the methanol-inducible promoter. Zeocin-resistant transformants were then screened for protein expression. CDH activity was successfully detected in the supernatant after induction, indicating the functional expression of the α-factor signal sequence. The recombinant CDH was secreted at high levels (0.3g/L after 96 h of induction) and was purified using the His-Bind resin.

**FIGURE 2:**
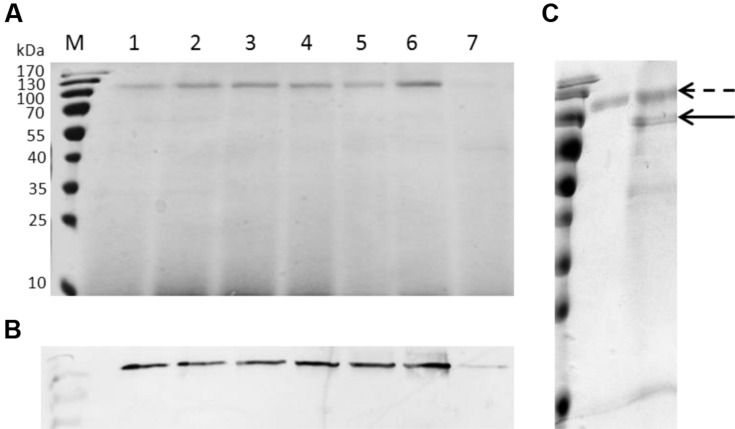
**SDS-PAGE, Western blotting analysis and deglycosylation of recombinant CDH. (A)** SDS-PAGE. Lane M: protein marker, lane 1–7: the culture supernatants of GS115 harboring pWM78 at 48, 60, 72, 78, 84, 96, 24 h after induction, respectively. Equal amounts of 10 μL samples were loaded for all lanes. At the early stage of induction (lane 7, 24 h after induction), the amount of protein was 0.2 μg, while at the late stage of induction (lane 6, 96 h after induction), the amount of protein was 2.95 μg. **(B)** Western blotting analysis using anti-His antibodies. Lanes are the same as A. **(C)** Deglycosylation of recombinant CDH showed a loss of approximately 30 kDa. Dotted arrow indicates CDH before deglycosylation while solid arrow indicates CDH after deglycosylation.

The purified CDH displayed a relative molecular weight around 110 kDa appeared on SDS-PAGE (**Figure 2A**). Western blot analysis confirmed the protein was CDH (**Figure 2B**). The protein sequence for possible *N*-glycosylation sites (Asn-Xaa-Ser/Thr) was checked with NetNGlyc 1.0 and six Asn-Xaa-Ser/Thr sites in the sequence were predicted to be *N*-glycosylated. Following deglycosylation of CDH, a band corresponding to its theoretical molecular weight was observed (**Figure 2C**). The purified CDH exhibited great affinity for cellobiose, with a *K*_M_ value of 8 μM and *k*_cat_ value of 14.92 s^-1^.

### Synergistic Effects between the Purified CDH with Cellulase of *T. reesei* QM9414

It has been well known that the expression levels of cellulase genes are different during the time-course cultivation. In order to assess the influence of CDH addition on the enzymatic hydrolysis of cellulase from *T. reesei* QM9414 at different cultivation time, the filter paper activity (FPA), endoglucanase (CMCase) and β-glucosidase activity were measured over a range of time points in the presence of 0, 10, and 50 μg recombinant CDH.

**FIGURE 3:**
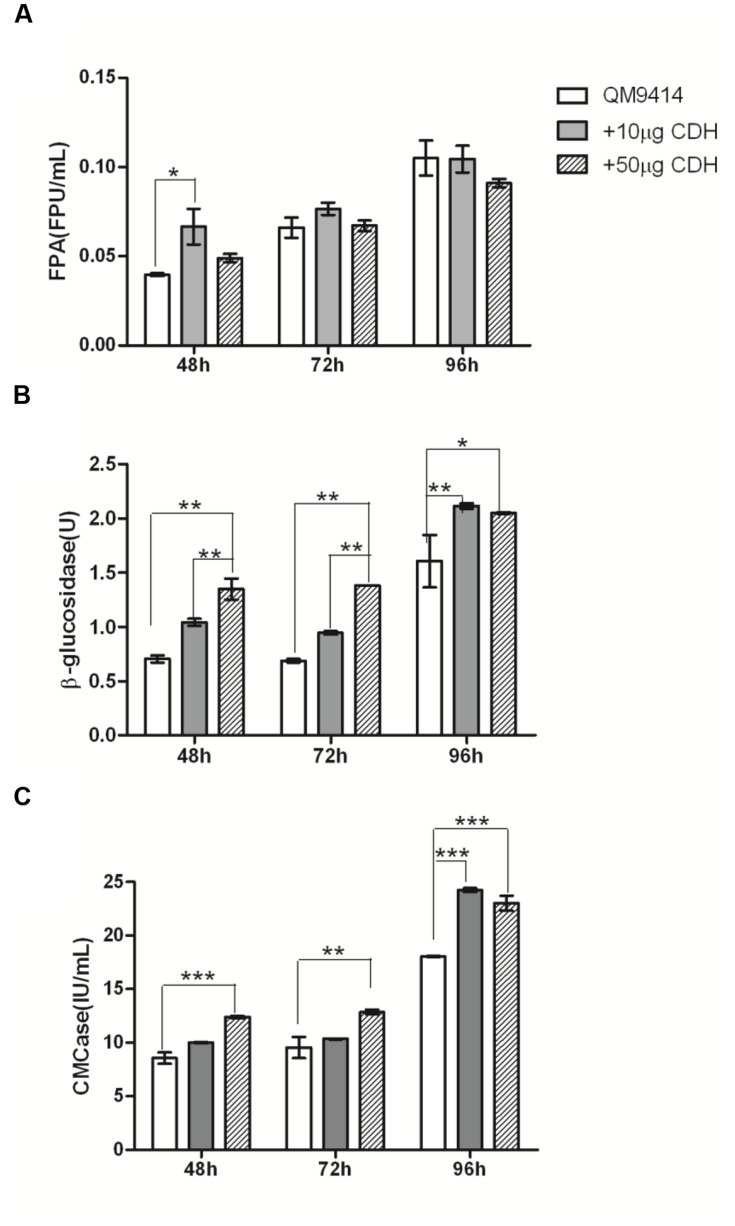
***In vitro* synergistic effects between the purified CDH and cellulase of *T. reesei* QM9414.** FPU activities **(A)**, β-glucosidase activities **(B)**, and CMCase **(C)** from the culture supernatants of *T. reesei* QM9414 for different times as indicated, in the presence of 0 μg (QM9414), 10 μg (+10 μg CDH) or 50 μg (+50 μg CDH) purified CDH. ^∗^ *P* < 0.05, ^∗∗^*P* < 0.01, ^∗∗∗^*P* < 0.001.

In the presence of 10 μg CDH, a significant increase in FPA activity (approximately 67.7%, *P* < 0.05) was observed in the culture supernatant of 48 h cultivation (**Figure 3A**), whereas in the presence of 50 μg CDH, the increase was not significant (~23.73%, *P* > 0.05). For the culture supernatants of 72 and 96 h cultivation, the changes of FPA activity were slightly increased in the presence of 10 μg CDH. In 96 h culture supernatant, the FPA activity decreased in the presence of 50 μg CDH. As for CMCase, the addition of 10 μg CDH caused approximately 8–34% increased activity at different time points (**Figure 3C**). When loading 50 μg CDH it was increased to approximately 27–45%. β-glucosidases seem to be more influenced by CDH addition. In the presence of 10 μg CDH, β-glucosidase activities were approximately 48, 37.5, and 32% higher in the culture supernatant of 48, 72, and 96 h cultivation (**Figure 3B**), respectively. As expected, further increasing CDH addition to 50 μg increased approximately 91 and 100% β-glucosidase activity in the culture supernatant of 48 and 72 h cultivation. In the culture supernatant of 96 h cultivation, 50 μg CDH addition did not significantly further increase either CMCase or β-glucosidase compared to the enzyme dosage of 10 μg CDH supplementation.

It indicates that the influence of CDH supplementation on cellulase not only depends on the CDH dosage, but also on the cultivation times of *T. reesei* QM9414, which correspond to different dosage and composition of cellulase mixture.

In the presence of 10 μg Ag-Bgl, approximately 15.2–17.6% increase in FPA activity were observed in the culture supernatants of 48 and 72-h cultivations (Supplementary Figure S1). For the culture supernatants of 96-h cultivation, the FPA activity were increased 9% in the presence of 10 μg Ag-Bgl. Consideration with the 0.038% residue glucose in the culture supernatant of 48-h point, the limited synergism effects between β-glucosidases and cellulase at 48-h point were due to the product inhibition of β-glucosidase by glucose. As for CMCase, the addition of 10 μg Ag-Bgl or Tm-BglA showed similar increased activity with CDH at different time points.

### *In Vivo* Synergistic Effects between Heterologous CDH and Native Cellulase in *T. reesei* QM9414

As the results showed above, the addition of recombinant CDH to a reaction mixture containing *T. reesei* QM9414 cellulase increased FPA, CMCase, and β-glucosidase activities. To discover how the synergy is functional *in vivo*, we then expressed the *cdh* in *T. reesei* QM9414 and evaluated the *in vivo* synergistic effects between CDH and cellulase. The promoter of the *cbh2* gene of *T. reesei* was used and the plasmid pWM78 (**Figure 1B**) was constructed as described in Materials and Methods. The transformant with the best performance was named as cdh:: QM9414. To guarantee comparisons between the native and mutant strains, we examined the growth pattern that was represented by dry weight biomass. The cdh::QM9414 showed retarded but no significant difference in growth when comparing to the parental QM9414 (Supplementary Figure S2).

**FIGURE 4:**
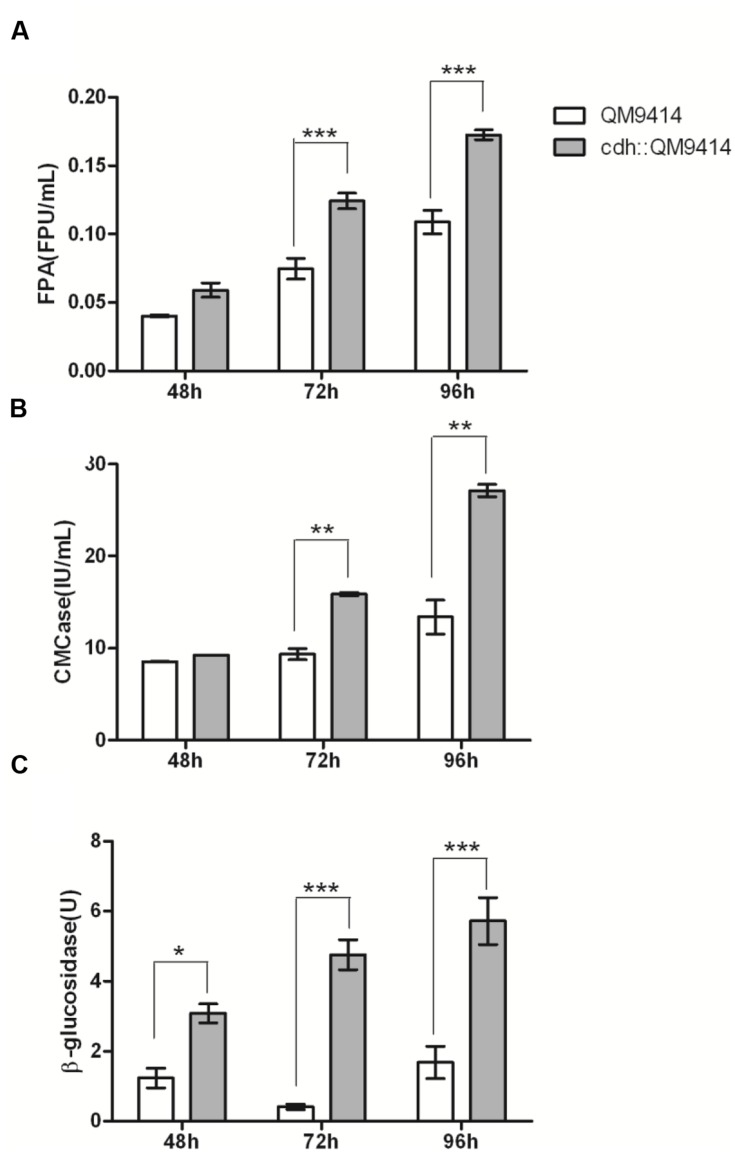
***In vivo* synergistic effects between CDH and cellulase of *T. reesei* QM9414.** FPA activities **(A)**, CMCase activities **(B)**, and β-glucosidase activities **(C)** from the culture supernatants of *T. reesei* QM9414 and cdh::QM9414 for different times as indicated. Error bars are represented from three biological replicates. ^∗^*P* < 0.05, ^∗∗^*P* < 0.01, ^∗∗∗^*P* < 0.001.

The cellulase activity (FPA, CMCase, and β-glucosidase) were measured from the culture mediums of the wild-type and the mutant strains and the results were shown in **Figure 4**. The cdh::QM9414 had higher cellulase activities compared with the parental QM9414. The FPA activities were 47–59% higher at different cultivation time points. β-glucosidase activities were increased considerably by 10.5 and 2.4 times while CMCase activities were increased 70 and 100% at 72 and 96 h, respectively.

**FIGURE 5:**
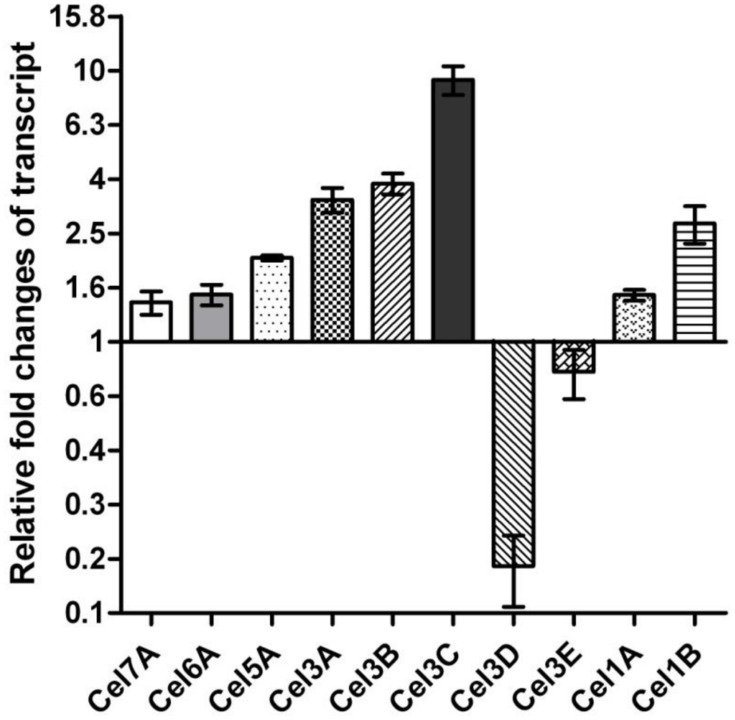
**Transcriptional changes of the selected genes in *cdh*-expressing strain compared to parental QM9414**.

The vast majority of the proteins in the culture supernatant of *T. reesei* is cellulase, especially three major cellulase components (CBH I, CBH II, and EG II). To determine whether the increased cellulase activity was also accompanied with the changes in cellulase expression level compared with parental QM9414, we analyzed the extracellular proteins of the *cdh*-expressing and the native QM9414 strains by SDS-PAGE (Supplementary Figure S3), and the secreted proteins were quantified by Bradford assay. Through the whole cultivation process the expression of *cdh* enriched the cellulase production compared with QM9414. This did not correlate with the growth profile (Supplementary Figure S2). Bradford assay showed the increase was 73% (*P* < 0.001) higher at 48 and 26.4% (*P* < 0.001) higher at 96 h. The BGL1 production, in particular, was remarkably enriched in the *cdh*-expressing strain. It is commonly accepted that the formation of cellulase is regulated at transcriptional level. This observation motivated us to investigate if the mRNA level of *bgl1* gene was indeed positively elevated. As the maximum increase of β-glucosidase activity was observed in 72 h, and the enhancement of BGL1 production was obvious in 48 h, we detected the mRNA levels in 48 h. Total RNA was extracted from mycelia after 48 h growing, from which cDNA was prepared and subjected to qRT-PCR (**Figure 5**). In the *cdh*-expressing strain cdh:: QM9414, mRNA level for *bgl1* gene was 2.3 times higher than level in native *T. reesei* QM9414. This is consistent with the protein levels as judged on SDS-PAGE gel (Supplementary Figure S3), but much lower than the 10.5-time increase observed in β-glucosidase activity (**Figure 4C**). This suggested that the enhancement of cellulase activity caused by CDH is not only due to its indirect effects on inducement of enzyme production, but also a functional synergism with cellulase during hydrolysis.

Eleven β-glucosidases have so far been characterized or predicted in *T. reesei* according to the predictions in the genome database (*T. reesei* v2.0) and CAZy database (Martinez et al., 2008). Besides the *bgl1*, we also wondered whether there is an increased up-regulation of other β-glucosidase genes of cdh::QM9414. We further detected relative mRNA levels of other six β-glucosidase (CEL1A, CEL1B, CEL3A, CEL3B, CEL3C, CEL3D, CEL3E) genes with qRT-PCR. Four of the six genes displayed the increased transcript levels. Relatively high levels of expression of these β-glucosidase genes in *cdh*-expressing strain implied that it might be caused by cellobiose degradation.

### *In Vivo* Synergistic Effects between CDH and Cellulase with Cellulose as the Carbon Source

As the results showed above, the synergism between CDH and cellulase was observed *in vivo* in fermentation process with lactose as the soluble carbon source. To get a better insight into the synergistic effects on natural substrates, we examined the performance of the *cdh* transformant strain with cellulose as the carbon source. The cdh::QM9414 and the parental QM9414 were pre-cultured in MA media supplemented with 0.45% (w/v) lactose and resuspended in MA media containing 1% (w/v) microcrystalline cellulose as the sole carbon source. As the insoluble cellulose particles were enwrapped with mycelia, it is hard to weigh the dry biomass. Thus the amount of intracellular protein content was measured for monitoring cell growth. No difference was observed on cellulose at 48 and 72 h (Supplementary Figure S4A). At 96 h, the intracellular protein of cdh::QM9414 decreased about 50% compared to the parental QM9414 strain.

The extracellular protein content was determined by Bradford assay and the expression profile was analyzed on SDS-PAGE (Supplementary Figure S5). When growing on cellulose, the enzyme production of cdh::QM9414 was increased by 10–25% at different time points compared to QM9414 (Supplementary Figure S4B). The abundance of the expressed CDH and BGL1 proteins are much lower than the three major cellulase components (Supplementary Figure S5). With cellulose as the carbon source, the cdh::QM9414 also secreted CDH protein into the culture medium, and enhanced the BGL1 production. Compared with the expression profile on lactose as the carbon source (Supplementary Figure S3), the cellulase was notably induced by cellulose. In addition, although the *cdh* was controlled under the *cbh2* promoter, the protein expression level for CDH production is far behind that achievable by native CBH II (Supplementary Figure S5).

**FIGURE 6:**
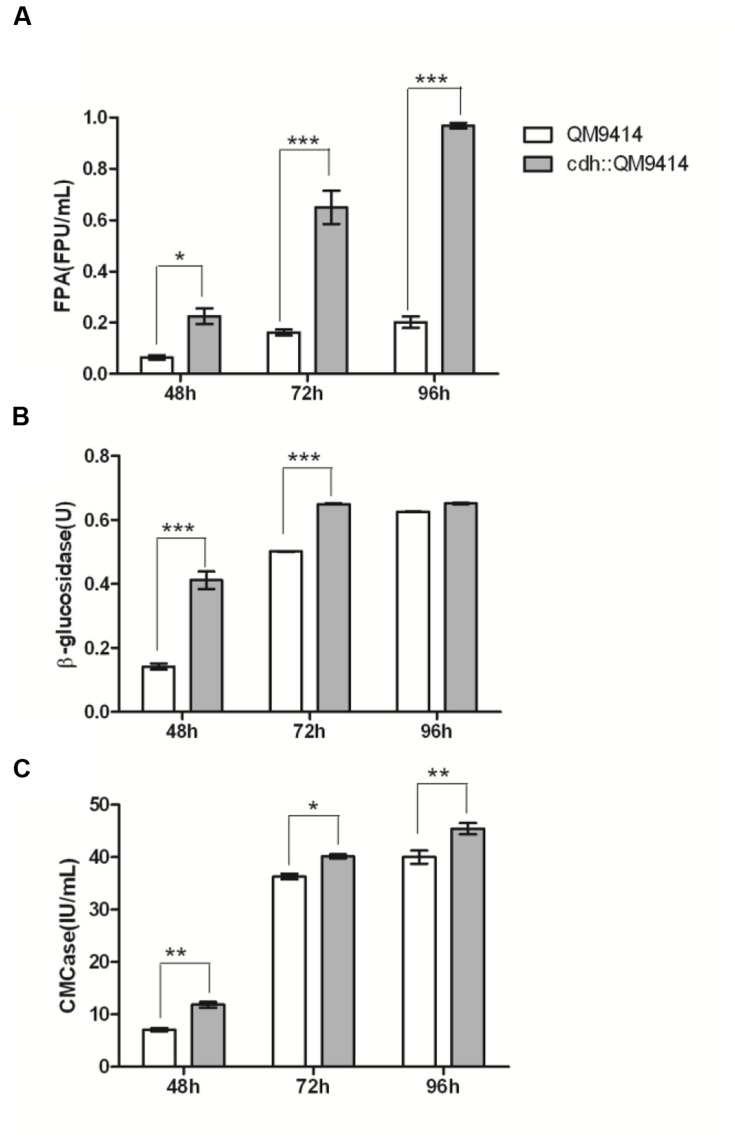
***In vivo* synergistic effects between CDH and cellulase of *T. reesei* QM9414 with cellulose as carbon source.** FPA activities **(A)**, β-glucosidase activities **(B)**, and CMCase activities **(C)** from the culture supernatants of *T. reesei* QM9414 and cdh::QM9414 with cellulose as carbon source for different times as indicated. Error bars are represented from three biological replicates. ^∗^*P* < 0.05, ^∗∗^*P* < 0.01, ^∗∗∗^*P* < 0.001.

Different from the synergism observed with lactose as the carbon source, the most significant synergism between CDH and cellulase components was observed in FPA catalysis rather than β-glucosidase (**Figure 6**). The FPA activities of cdh::QM9414 were increased about 2.5–3.8 times compared to QM9414 on cellulose. This was not corresponding with the protein expression profile on SDS-PAGE. Therefore, there is also a functional synergism between CDH and cellulase during hydrolysis of cellulose. The CMCase activities were raised sharply at 48 h on cellulose, but slowed down at 72 and 96 h. β-glucosidase activities increased 190 and 39% at 48 h and 72 h, respectively, whereas there is no significant change compared to parent strain QM9414 at 96 h (**Figure 6**). It’s confirmed that a *cdh*-expressing *T. reesei* strain has a synergistic effect upon the overall cellulase activity on pure microcrystalline cellulose.

## Discussion

Currently cellulosic biofuels production receives massive attention (Fairley, 2011). However, efficient cellulase production has been a hurdle to be overcome. Although the model cellulolytic organism *T. reesei* is presently used to produce cellulase mixtures, the productivity and the performance for some prohibitive substrates require further improvement for such industrial enzyme-based production system to make it economically viable (Klein-Marcuschamer et al., 2012). Enzymatic hydrolysis of cellulose is a complex process, where the last step is a critical step and involves a catalytic reaction of β-glucosidase with cellobiose as the substrate, a strong inhibitor of both cellobiohydrolases and endocellulases. The native β-glucosidases produced by *Trichoderma* are not enough to reduce cellobiose inhibition and additional β-glucosidase supplementation is necessary. Research efforts on cellulase performance have recently widened scope to explore new types of enzymes that substantially improve cellulose degradation. Synergistic interactions of different enzymes or non-enzymatic proteins result in more efficient cellulose degradation. The oxidative enzyme AA9 (formerly GH61, reclassified as family AA9 by CAZy, Lombard et al., 2014) could promote the efficiency of classical cellulases (Horn et al., 2012), and the oxidative enzymes are now present in commercially available cellulase preparations such as CTec2 to improve the conversion yield (Cannella et al., 2012). CDH was also reported as oxidative enzyme. In this study, we explored the synergistic effects between cellulase and CDH, and discuss the promising of combining CDH with the current β-glucosidase and other available addition enzymes to enhance cellulase performance.

Although the physiological function of CDH in cellulose biodegradation has not been elucidated yet, some experimental observations related to a possible function have been made: CDH enhances *P. chrysosporium* CBH I activity by oxidizing the inhibitor cellobiose to cellobionolactone. In the current study, we confirmed the synergism between the purified CDH and cellulase from *T. reesei in vitro* and further constructed a *cdh* overexpressed stain and investigated the synergism *in vivo*. Based on both *in vitro* and *in vivo* experiments, the synergism was illustrated with FPA, CMCase and β-glucosidase activities. Besides the synergism between CDH and CBH I that observed previously, we found that CDH increased the β-glucosidase activity remarkably. It implies that CDH and β-glucosidases work synergistically in cellobiose metabolism. However, the comparison of synergism effects between CDH and cellulase with synergism effects between β-glucosidases and cellulase indicates different functions of CDH and β-glucosidases during the enzymatic hydrolysis process. The supplemention of β-glucosidases mainly balances the deficiency of low amount of β-glucosidases in *T. reesei* cellulase and releases the inhibition of the accumulation of cellobiose as the end product. And β-glucosidases are also sensitive to the product inhibition by glucose. Combined with the transcriptional analysis of *bgl* and *cdh* in relation to cellobiose metabolism (Yoshida et al., 2004) some light could be thrown on how intracellular expression of CDH lead to an increase in the transcript levels of β-glucosidase. Transcriptional analysis in *T. reesei* has revealed that the transcription of *bgl* gene is repressed by cellobiose. It is reasonable to assume that the expression of CDH decrease the cellobiose concentration and relieve inhibition of β-glucosidase transcription. However, a more systematic insight into the mechanism with the exploration of the synergistic dynamics and interactions between CDH and CBH I, CBH II, EGs, and β-glucosidases need to be elucidated in future.

The diversity of β-glucosidases transcription suggests that these enzymes are regulated and functioning in different ways. Although BGL1, CEL3B, and CEL3E all belongs to the family GH3 and are predicted to be secreted according to the signal sequence prediction (SignalP 4.0), but their transcriptions are different. CEL3C and CEL3D, which are in the same protein homology cluster showed similar results. Both amino acid sequences showed no predicted transmembrane domain or signal peptide, which in turn means that both CEL3C and CEL3D work in the cytoplasm. Notably, CEL3C was up-regulated with 9.27-fold changes. This result confirmed the different functional subgroups of GH3 family determined according to phylogenetic analysis (Hakkinen et al., 2012) (Supplementary Figure S6).

The expression of *cdh* was also found to cause a slightly increased mRNA level of *cbh1*, *cbh2*, and *eg2* than those in the native *T. reesei*. One possible explanation is that the expression of the different cellulase genes has been reported to be coordinative. Continuing interest and efforts in understanding the regulating of cellulase has revealed that the expression of *cbh1*, *cbh2*, and *eg2* genes are subjected coordinatly to both positive (Xyrl, Ace2, Hap2/3/5) and negative (Crel, Acel) regulation (Schmoll and Kubicek, 2003). The recent exploration of the *bgl1*, *cel1a*, and *cel1b* in the induction of cellulase genes by lactose in *T. reesei* demonstrated that *cbh1* gene expression is co-regulated with β-glucosidase genes (Xu et al., 2014). The intracellular β-glucosidase also acts on activating XYR1, which in turns regulates the *cbh1*, *cbh2*, and *eg2* gene expression.

Cellobiose dehydrogenase oxidizes cellobiose to cellobionolactone, which is subsequently hydrolyzed to cellobionic acid. In *P. chrysosporium*, CDH increased CBH I activity by releasing competitive inhibition on CBH I by oxidization of cellobiose to cellobionolactone, however, it is reported that cellobionolactone was more inhibitory to the *T. viride* CBH I (Igarashi et al., 1998). On the other hand, though cellobionic acid is known to be less inhibitory for cellulases than cellobiose (Igarashi et al., 1998), it could be hydrolyzed by β-glucosidase at a very low rate (almost 10-fold lower than for cellobiose), and the formed gluconic acid showed stronger product inhibition than glucose (Cannella et al., 2012). In this study, particularly shown in **Figure 3A**, when extra CDH (50 μg vs. 10 μg) was added to cellulase mixture, a decrease of the FPA activity was observed. The mixture of cellulase and CDH will require further tuning to obtain the optimal radio and enzyme dose to maximize their cellulolytic synergism function.

The CDH expression in *T. reesei* needs to be optimized further. In consideration of the conclusion derived from **Figure 3**, we constructed the plasmid with the *cdh* under the relatively mild *cbh2* promoter instead of commonly used strong promoter *cbh1*. Unexpectedly, with the same promoter *cbh2*, the protein expression level for *cdh* production is far behind that achievable by native CBH II. Thus the CDH expression needs to be enhanced by using other promising promoters such as *cbh1*. Here we chose QM9414 as the parental strain and next this strategy could also be applied to the industrial strains. One of the highest cellulase producers is RUT-C30 with a titer of 30 g/L and a number of industrial strains are derived from this mutant (Seidl et al., 2008; Kubicek et al., 2009; Schuster et al., 2012). Further exploration of the synergism in RUT-C30 or other derived industrial strains should be very important. Another notable aspect is the development of new pretreatment procedures for the improved cellulose digestibility. Various types of pretreatment procedures result in different cellulose accessibilities. Thus, in real industrial processing, the impact of adding CDH to cellulase cocktails is likely to vary depending on the substrates. Our results only showed the synergism with soluble lactose and pure microcrystalline cellulose as carbon source. More work is needed to further refine the synergism with more complex carbon sources.

In summary, this study demonstrated synergistic effects between CDH and *T. reesei* cellulase components including FPA, CMCase, and β-glucosidase with both *in vitro* and *in vivo* observations. The results not only confirmed the synergism between CDH and *P. chrysosporium* CBH I that previously observed, but also suggested that CDH increased the β-glucosidase activity remarkably. It implies that CDH and β-glucosidases work synergistically in cellobiose metabolism. It elucidates a possible mechanism for diminishing the cellobiose inhibition on cellulases by CDH. An integration of different strategies including CDH expression shows a promising potential for improving cellulose efficiency and thus promoting cellulosic biofuels production.

## Author Contributions

MW and XL designed and coordinated the study and wrote the manuscript. MW carried out the experiments and analyzed the results. Both authors read and approved the final manuscript.

## Conflict of Interest Statement

The authors declare that the research was conducted in the absence of any commercial or financial relationships that could be construed as a potential conflict of interest.
