# Supplementary material for: Exploring the Synergy between Cellobiose Dehydrogenase from Phanerochaete chrysosporium and Cellulase from Trichoderma reesei
Source: Front Microbiol. 2016 Apr 29;7:620. doi: 10.3389/fmicb.2016.00620 (PMC4850161; doi:10.3389/fmicb.2016.00620)
Supplement: Supplementary file 1 [file Data_Sheet_1.DOCX]

**Supplementary information**

**Table S1 Primers and plasmids used in this study**

| **Primer** | | **Sequence(5'to3')** | **Remarks** | | **Amplification**  **Efficiency(%)** | |
| --- | --- | --- | --- | --- | --- | --- |
| mcdh-Ep1 | | gggagaattccagagtgcctcacagtttacc | m-cdh Forward primer(EcoRI) | |  | |
| mcdh-Xbp2H | | tttttctagaggacctcccgcaagcgcga | m-cdh Reverse primer(XbaI) | |  | |
| CBH2-001Nd | | gggcatatgattgtcggcattctc | cbh2 Forward primer(NdeI) | |  | |
| CBH2-RXh | | atttctcgagcaggaacgatggg | cbh2 Reverse primer(XhoI) | |  | |
| cbh1RP-F | | tggcaacgagttctctttcg | cbh1 qRT-PCR Forward primer | | 98.52 | |
| cbh1RP-R | | tgttggtgggatacttgct | cbh1 qRT-PCR Reverse primer | |  |  |
| cbh2RP-F | | ccattcgtcaaattgtcgtg | cbh2 qRT-PCRForward primer | | 112.65 | |
| cbh2RP-R | | tgactgagcattggcacac | cbh2 qRT-PCRReverse primer | |  |  |
| eg2RP-F | | tcatcaactccacccacg | eg2 qRT-PCRForward primer | | 99.88 | |
| eg2RP-R | | agttgtttgagccggtga | eg2 qRT-PCRReverse primer | |  |  |
| cel3ARP-F | | cggtacgagttcggctat | bgl1 qRT-PCRForward primer | | 104.59 | |
| cel3ARP-R | | ggaacagatcactcgggcct | bgl1 qRT-PCRForward primer | |  |  |
| cel3BRP-F | | atgaagacgttgtcagtgtttg | cel3B qRT-PCRForward primer | | 97.77 | |
| cel3BRP-R | | aactccttagcttgggcata | cel3B qRT-PCRReverse primer | |  |  |
| cel3CRP-F | | atggctgatattgatgttgag | cel3C qRT-PCRForward primer | | 102.72 | |
| cel3CRP-R | | cagggacgccattgaaga | cel3C qRT-PCRReverse primer | |  |  |
| cel3DRP-F | | tggctacaagttctacgacaa | cel3D qRT-PCRForward primer | | 93.42 | |
| cel3DRP-R | | tggctactgtgggctggt | cel3D qRT-PCRReverse primer | |  |  |
| cel3ERP-F | | atgcggctgtgtgacttatc | cel3E qRT-PCRForward primer | | 105.49 | |
| cel3ERP-R | | gcccacggtccatttcct | cel3E qRT-PCRForward primer | |  |  |
| cel1ARP-F | | atgttgcccaaggactttcag | bgl2 qRT-PCRForward primer | | 90.2 | |
| cel1ARP-R | | acgagccgtcggcgatcttg | bgl2 qRT-PCRForward primer | |  |  |
| cel1BRP-F | | ctttgcgtgggcgttgct | cel1B qRT-PCRForward primer | | 86.6 | |
| cel1BRP-R | | ttatgccgccactttaaccct | cel1B qRT-PCRReverse primer | |  |  |
| actinRP-F | | tgagagcggtggtatccacg | actin qRT-PCRForward primer | | 79.04 | |
| actinRP-R | | gtaccaccagacatgaca | actin qRT-PCRReverse primer | |  |  |
| **plasmid** | **Genotype/phenotype** | | | **Reference** | |  |
| pSMZ1 |  | | | From Prof.Kubicek | |  |
| pc-cdh | pUC ori, Ap^r^, P.chrysosporium cdh cloned into pUC57 | | | This study | |  |
| pWM78 | pUC ori, Ap^r^, P.chrysosporium cdh cloned into pSMZ1 | | | This study | |  |
| pWM79 | pUC ori, Zeocin^r^, P.chrysosporium cdh cloned into pPICZaA | | | This study | |  |
| pWM64 | pUC ori, Ap^r^, fragment of actin for qRT-PCR cloned into pMD 18-T | | | This study | |  |
| pWM65 | pUC ori, Ap^r^, fragment of bgl1 for qRT-PCR cloned into pMD 18-T | | | This study | |  |
| pWM68 | pUC ori, Ap^r^, fragment of bgl2 for qRT-PCR cloned into pMD 18-T | | | This study | |  |
| pWM66 | pUC ori, Ap^r^, fragment of cel3B for qRT-PCR cloned into pMD18-T | | | This study | |  |
| pWM70 | pUC ori, Apr, fragment of cel3C for qRT-PCRcloned into pMD 18-T | | | This study | |  |
| pWM71 | pUC ori, Apr, fragment of cel3D for qRT-PCRcloned into pMD 18-T | | | This study | |  |
| pWM67 | pUC ori, Apr, fragment of cel3E for qRT-PCRcloned into pMD 18-T | | | This study | |  |
| pWM69 | pUC ori, Apr, fragment of cel1B for qRT-PCRcloned into pMD 18-T | | | This study | |  |

**
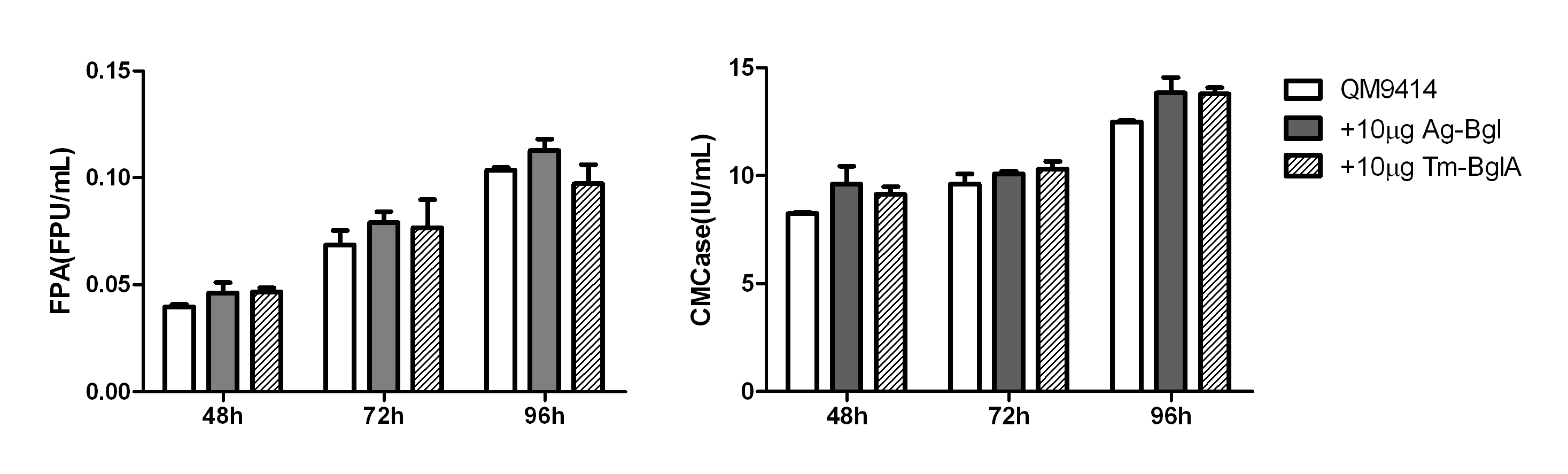
**

**Figure S1 Synergistic effects between β-glucosidase and cellulase of *T. reesei* QM9414.**

FPU activities and CMCase from the culture supernatants of *T. reesei* QM9414 for different times as indicated, in the presence of 10 μg β-glucosidase from *Aspergillus niger* (+10μg Ag-Bgl), or 10 μg recombinant β-glucosidase from *Thermatoga maritima* (+10μg Tm-BglA).


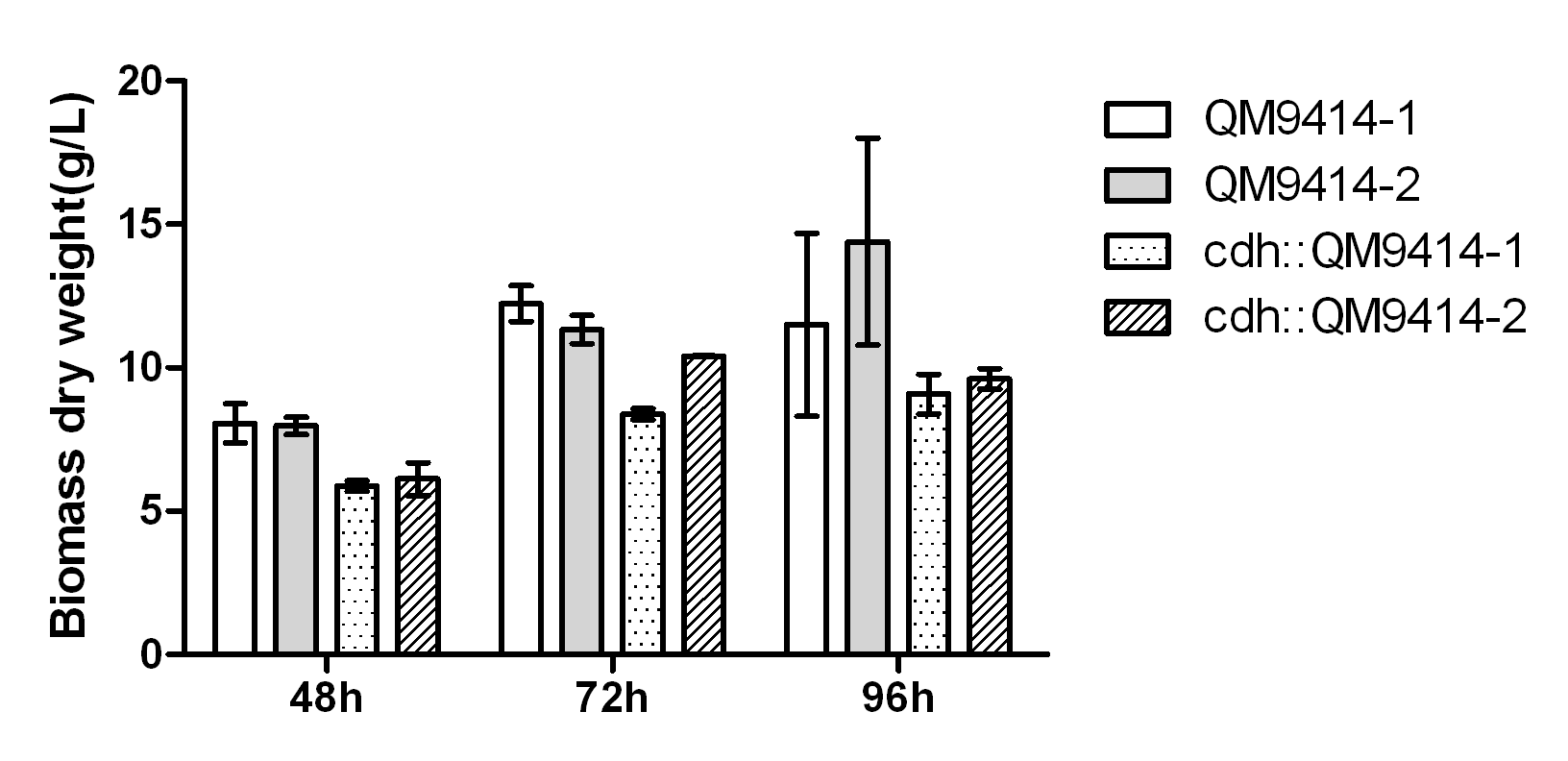


**Figure S2 Growth profile of *T. reesei* QM9414 and cdh::QM9414.**


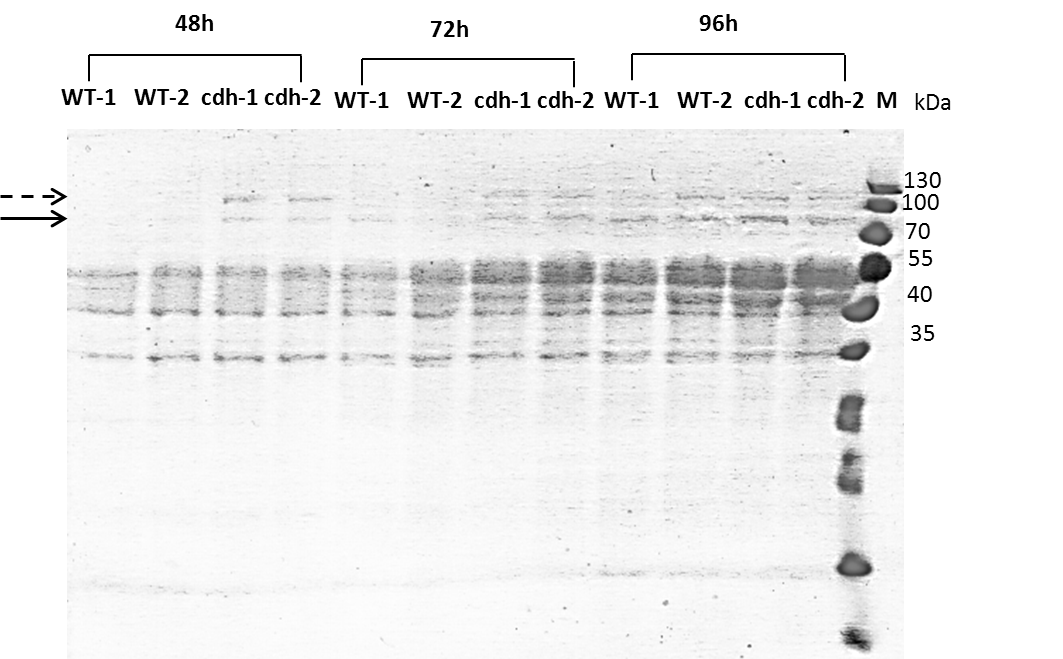


**Figure S3 SDS-PAGE analysis of the culture supernatants of *T. reesei* QM9414 and cdh::QM9414.**

WT, QM9414 parental strain; cdh, cdh::QM9414. Lane M: protein marker, -1, -2 represent two replicates. Dotted arrows indicate heterologous expressed CDH in *T. reesei*, and solid arrows indicate β-glucosidase BGL1. The amounts of protein that were loaded on lane1-12 were 4.56 μg, 6.21μg, 4.66 μg, 6.54 μg, 7.56 μg, 8.72 μg, 7.87 μg, 7.97μg, 9.36 μg, 9.77 μg, 12.12 μg and 12.53 μg, respectively.


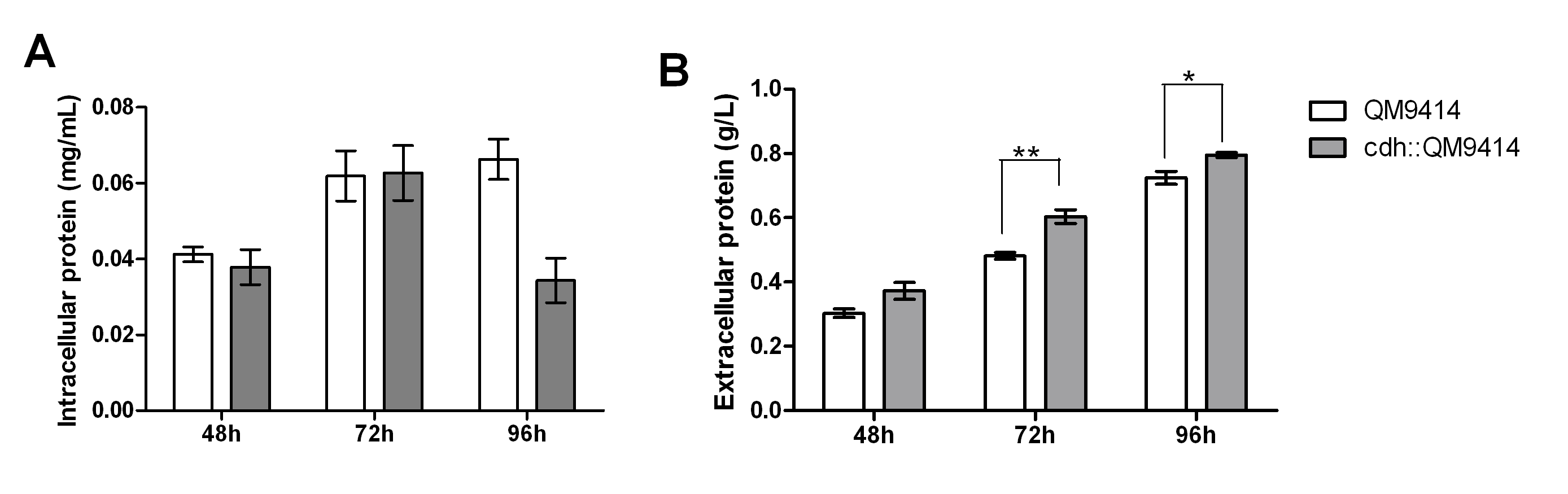


**Figure S4 Intracellular and extracellular protein concentrations of *T. reesei* QM9414 and cdh::QM9414 on cellulose.**

The amount of protein was quantified by the Bradford protein assay kit (Beyotime) with bovine serum albumin (BSA) as standard. **P*<0.05, ***P*<0.01, *** *P*<0.001.

**A** When grows on cellulose, the insoluble cellulose particles were enwrapped with mycelia, it is hard to weigh the dry biomass. Thus the amount of intracellular protein content was measured for monitoring cell growth.


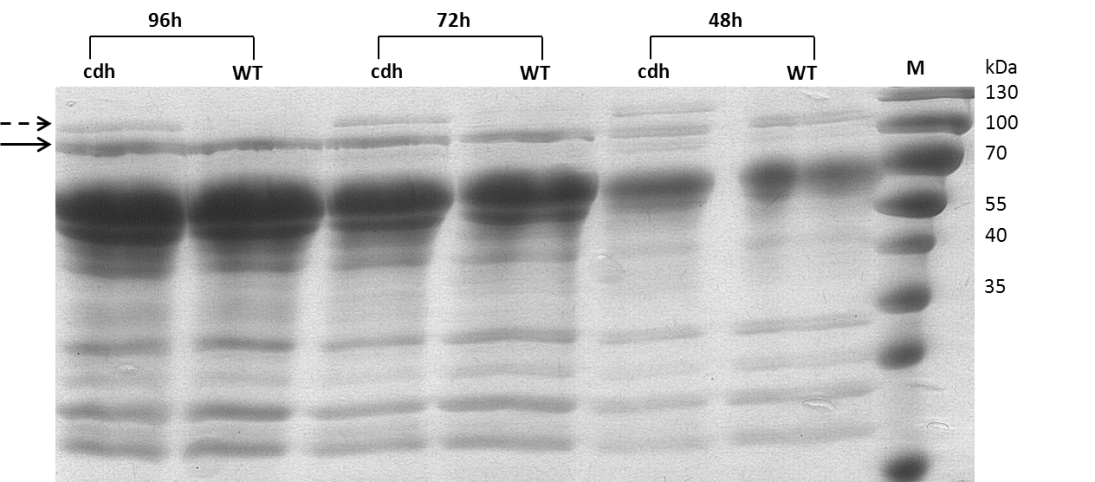


**Figure S5 SDS-PAGE analysis of** **the** **culture supernatants of *T. reesei* QM9414 and cdh::QM9414 on cellulose.**

WT, QM9414 parental strain; cdh, cdh::QM9414. Lane M: protein marker. Dotted arrows indicate heterologous expressed CDH in *T. reesei*, and solid arrows indicate β-glucosidase BGL1. The amounts of protein that were loaded on lane1-6 were 31.89 μg, 29.08 μg, 24.19 μg, 19.29 μg, 14.95 μg and 12.17 μg, respectively.


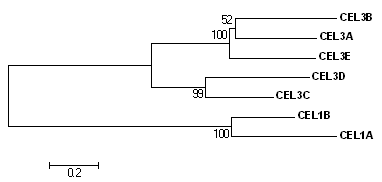


**Figure S6 Phylogenetic tree of certain β-glucosidases of *T. reesei***

The amino acid sequences of the seven proteins were aligned using Clustal X2.0. The phylogenetic tree was constructed using MEGA 5.1 via the neighbour-joining method.
